# Convention of Delegates from Medical Colleges

**Published:** 1855-04

**Authors:** 


### *Convention of Delegates from Medical Colleges.*

ONE of our exchanges, we think the Nashville Journal, suggests that the next meeting of the American Medical Association should recommend a "National Convention of delegates from the Medical

Colleges" of the whole Union, to consider practically the subject of medical education.

Such a recommendation was made at the meeting of the Association in Boston, in 1849. The proposition was made by a committee, consisting of Alexander H. Stevens, of N. Y; George B. Wood, of Philadelphia; and Jonathan Knight, of New Haven; and unanimously adopted. The time recommended for the meeting was a few days before the Annual Meeting of the Association in Cincinnati, in 1850. But the Colleges paid no heed to it whatever. Perhaps a renewal of the recommendotion would meet with success. We are in favor of trying it, for we are confident that such a meeting would result in much good.

